# *In vitro* damage of *Candida albicans* biofilms by chitosan

**DOI:** 10.3892/etm.2014.1839

**Published:** 2014-07-11

**Authors:** YU PU, AIBO LIU, YUQIANG ZHENG, BIN YE

**Affiliations:** 1 Department of Pathogenic Biology, Chongqing Medical University, Chongqing 400016, P.R. China; 2 Department of Medicine Laboratory, Children’s Hospital of Chongqing Medical University, Chongqing 400014, P.R. China; 3 Research Center for Molecule Medicine and Tumor, Chongqing Medical University, Chongqing 400016, P.R. China

**Keywords:** *Candida albicans*, chitosan, biofilms

## Abstract

With the increasing usage of indwelling medical devices in clinical practice, the frequency of fungal infections has increased, such as that of *Candida albicans* (*C. albicans*). Biofilms, a protected niche for microorganisms, are resistant to a range of current antifungal agents. Chitosan is a polyatomic biopolymer with advantageous biocompatibility, biodegradation, nontoxicity and antibacterial properties. To investigate the inhibitory effect of chitosan on biofilms formed by *C. albicans*, cell viability, 2,3-bis(2-methoxy-4-nitro-5-sulfophenyl)-2H-tetrazolium-5-caboxanilide reduction, and morphological assays, including fluorescence microscopy and scanning electron microscopy (SEM), were employed. As assessed by cell viability assay, chitosan showed significant inhibitory effects on the planktonic cells and the biofilm of *C. albicans* in a dose-dependent manner. Fluorescence microscopy and SEM assays confirmed that the chitosan-treated group showed delayed *C. albicans* biofilm formation with defect morphological features, due to the inhibitory effects of the vast majority of fungal cell growth. In conclusion, *C. albicans* biofilms were compromised by the treatment with chitosan, providing an alternative therapeutic strategy against the fungal biofilms in the medical devices.

## Introduction

*Candida albicans* (*C. albicans*), an opportunistic pathogen, is able to attack hosts that are immunocompromised or otherwise debilitated. With the increased use of immunosuppressive and cytotoxic drugs, antibiotic abuse and implanted devices, including urinary catheters and endotracheal tubes, the prevalence of fungal infections has also increased (1–3). *C. albicans* was the leading cause of pulmonary fungal infection in 2011 and the drug resistance rate demonstrates a tendency to increase year by year (4). A number of implanted devices, including intravascular or urinary catheters and endotracheal tubes, are associated with fungal infections, and a biofilm may be detected on their surface (5–7). In recent years, emerging cases of *C. albicans* drug resistance have been primarily attributed to the formation of biofilms, with resistance increasing in conjunction with the maturation of the biofilm (8). Mature *Candida* biofilms consist of a complex three-dimensional structure of layers of yeast cells, hyphae and an abundant exopolysaccharide matrix (9,10). As natural resistance barriers of antifungal drugs, biofilms play a significant role in the invasion and dissemination of *C. albicans*.

Chitosan is a natural linear polyatomic biopolymer comprising N-acetyl-D-glucosamine and β-(1,4)-linked D-glucosamine. A previous study revealed that surfaces coated with chitosan were able to resist biofilm formation by bacteria and fungi *in vitro* (11). Chitosan is nontoxic to humans and exhibits excellent biocompatibility. The biopolymer is easily catalyzed to oligosaccharides by various biological enzymes, and is considered biodegradable (12); thus, can be absorbed by the body. Previous studies have demonstrated that chitosan not only has immunoregulatory efficacy, but also a naturally broad spectrum of antibacterial activity that enables the inhibition of biofilm formation (11,13,14).

Chitosan is an extensively studied biomacromolecule that is typically derived from chitin, a major component of crustacean shells. Due to their biocompatible and biodegradable properties, chitosan and its derivatives have been proposed for application in novel drug studies. The effect of chitosan on bacterial biofilms has been extensively reported; however, the aim of the present study was to investigate the susceptibility of *C. albicans* biofilms to chitosan, with an emphasis on determining the effects of chitosan on various biofilm growth phases and architectural organization.

## Materials and methods

### C. albicans

A *C. albicans* strain, previously isolated from a patient, was obtained from the Clinical Laboratory of the Children’s Hospital of Chongqing Medical University (Chongqing, China). The fungus was inoculated on a chocolate agar plate and grown at 37°C for 48 h. A single colony of the desired strain was inoculated into 8 ml Sabouraud dextrose broth (SDB; Sangon Biotech Co., Ltd., Shanghai, China) and incubated overnight at 37°C in a rotary shaker at 220 rpm.

### Chitosan susceptibility in C. albicans planktonic cells

Chitosan (Sigma-Aldrich, St. Louis, MO, USA) was solubilized in 0.2% acetic acid at a working concentration of 1% to pH 5.0–5.5. Serial two-fold dilutions of chitosan stock solutions in SDB were prepared in plastic centrifuges over a range of 0.0078–0.5% and stored at 4°C. A standard inoculum of 1v10^7^ cells [optical density (OD)_600_] from the overnight culture of the fungal strain was prepared prior to each experiment. Briefly, 100 μl *C. albicans* planktonic cells were mixed with 100 μl chitosan solution of various concentrations and incubated for 48 h at 37°C. Data were obtained from three independent experiments. For the positive control, *C. albicans* planktonic cells were incubated in the presence of 200 μl SDB without chitosan, while in the negative control, 200 μl SDB only was incubated in wells under otherwise identical conditions. The minimum inhibitory concentrations (MIC)_50_ for the biofilms and planktonic cells were defined as the minimum antifungal concentration that caused ≥50% fungal damage compared with the untreated controls.

### Measurement of chitosan susceptibility in biofilms using a 2,3-bis-(2-methoxy-4-nitro-5-sulfophenyl)-2H-tetrazolium-5- carboxanilide (XTT) reduction assay

*C. albicans* biofilm experiments were performed in untreated 96-well plates. The wells of the 96-well plates were incubated for various time periods (2, 8, 24 and 48 h) in 100 μl *C. albicans* suspension (1×10^7^ cells/ml) at 37°C. The medium in each well was removed at the indicated time points and the biofilms were washed twice with phosphate-buffered saline (PBS). Following the removal of PBS, 100 μl chitosan solution of various concentrations were added to one well of a 96-well plate and then incubated for additional 48 h at 37°C. A formazan salt-based XTT reduction assay was performed to assess the metabolic activity (15). All the tests were performed in duplicate and the average was calculated. Positive and negative controls were established as previously described. A total of 100 μl XTT-menadione solution, consisting of XTT (Sangon Biotech) salt solution (0.5 g/l in Ringer-Locke liquor) mixed with menadione (Sangon Biotech Co., Ltd.) solution (1 mM in acetone; Sigma-Aldrich), was added to each well. The 96-well plates were incubated for 2 h at 37°C. Colorimetric changes were scanned at 490 nm with a Varioskan^™^ Flash Multimode Reader (Thermo Fisher Scientific, Waltham, MA, USA).

### C. albicans biofilm formation with cover slips

Cover slips (0.8×0.8 cm) that were used for biofilm growth were soaked in concentrated sulfuric acid overnight. The following day, the concentrated sulfuric acid was washed with flowing water and the clean cover slips were immersed in 95% alcohol overnight. All the cover slips were washed three times with deionized water. For biofilm growth on the treated cover slips, as aforementioned, the cover slips were placed in 24-well plates and immersed in fetal bovine serum (FBS; Sigma-Aldrich, Beijing, China) at 4°C overnight. Following this pretreatment, the cover slips were washed with PBS (0.01 M) to remove the residual FBS. To ensure uniform biofilm formation, the cover slips were immersed in 1 ml standardized cell suspension (1×10^7^ cells/ml) and incubated for 90 min at 37°C. The cover slips were lifted carefully using tweezers and gently placed in each well of the 24-well plate containing 1.5 ml fresh SDB medium. Samples were incubated for various durations at 37°C.

### Susceptibility of the biofilms to chitosan

To evaluate the chitosan susceptibility of *C. albicans* cells grown in developing biofilms, the pretreated cover slips were immersed in 1 ml standardized cell suspension (1×10^7^ cells/ml) and incubated for 90 min at 37°C. The cover slips were lifted carefully using tweezers and gently placed in each well of the 24-well plate containing 1 ml fresh SDB medium. A 0.0625% chitosan solution was added to 24-well plates with cover slips and incubated for an additional 8, 24 and 48 h at 37°C.

### Fluorescence microscopy

Cover slips pretreated with biofilms were transferred to microscope slides and stained for 1 min with 50 μl Calcofluor White M2R [0.05% (vol/vol); Sigma-Aldrich] under minimal ambient light or in a darkroom. The biofilms were observed immediately under an ultraviolet (UV) range of 440 nm excitation and 500–520 nm emission wavelengths. Any superfluous fluorochrome on the cover slips was absorbed by the filter paper. The stained biofilms were examined under a fluorescence microscope.

### Scanning electron microscopy (SEM)

*C. albicans* biofilms were grown on pretreated cover slips in 24-well plates, as described previously. In the chitosan group, *C. albicans* biofilms were incubated in 0.0625% chitosan. For the positive control, *C. albicans* biofilms were incubated in the presence of 200 μl SDB without chitosan for 24 h. The cover slips with biofilms were subsequently washed three times with PBS and transferred to an additional 24-well plate containing 2.5% glutaraldehyde at 4°C. The samples were prepared using a regular method for electron microscopy examination (16) and viewed under an S-3000N scanning electron microscope (Hitachi High-Technologies, Tokyo, Japan). Two separate sets of culture were prepared.

### Statistical analysis

Statistical analyses were performed using SPSS 19.0 software (SPSS, Inc., Chicago, IL, USA). All data were tested for normality and transformed when necessary to meet the assumption of normal distribution. P-values were calculated by one-way analysis of variance and Fisher’s least significant difference test was conducted to determine differences among the test groups. P<0.05 was considered to indicate a statistically significant difference.

## Results

### MIC values of chitosan on C. albicans planktonic cells

**Figure 1:**
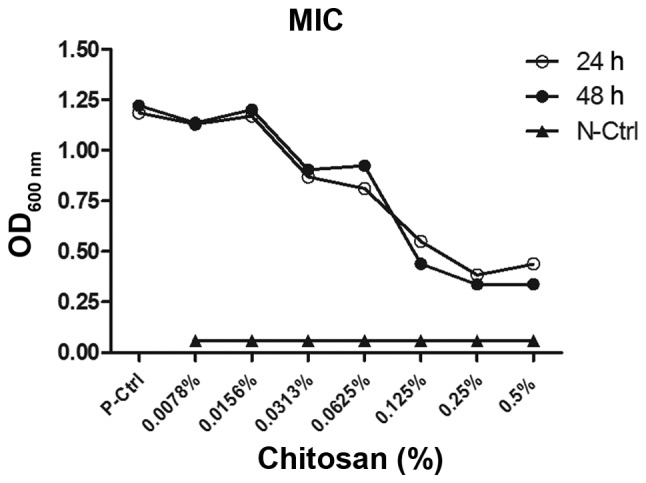
Growth curve of *Candida albicans* (*C. albicans*) planktonic cells with chitosan. Planktonic cells were co-incubated with various concentrations (0.5, 0.25, 0.125, 0.062, 0.0313, 0.0156 and 0.0078%) of chitosan for 24 and 48 h. Sabouraud dextrose broth served as a negative control. The line curves show that chitosan (>0.0313%) strongly inhibited the growth of *C. albicans*. Experiments were conducted three times, with similar results each time. OD, optical density; MIC, minimum inhibitory concentration.

Fig. 1 shows the results of the experiment assessing the effect of chitosan at various concentrations on *C. albicans* planktonic cell growth. Chitosan, at a concentration of >0.0313%, was shown to severely inhibit the activity of *C. albicans* planktonic cells grown for 24 h. The OD decreased significantly when compared with the positive control (P<0.05). When comparing the effect of chitosan over 24 and 48 h, the MIC of chitosan exhibited no statistically significant change on cell growth.

### C. albicans biofilm formation

**Figure 2:**
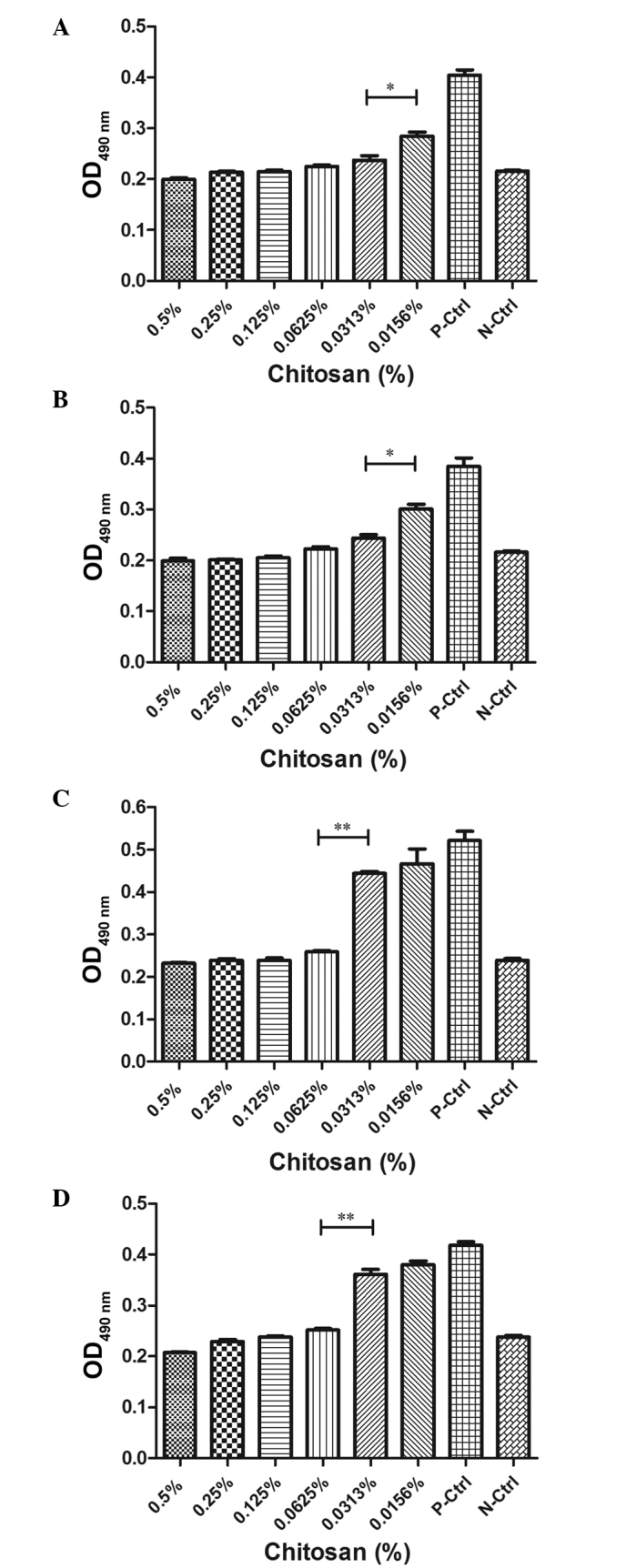
*Candida albicans* (*C. albicans*) biofilm development with chitosan resistance. The susceptibility of *C. albicans* biofilms, incubated for (A) 2 h, (B) 8 h, (C) 24 h and (D) 48 h, to various concentrations (0.5, 0.25, 0.125, 0.0625, 0.0313 and 0.0156%) of chitosan are represented as histograms. For the positive control, *C. albicans* planktonic cells were incubated without chitosan; for the negative control, sabouraud dextrose broth only was incubated in the wells. The OD values at the various stages of biofilm development were compared with those of the fungal cells and the negative control. Experiments were performed twice, with similar results obtained each time. OD, optical density.

An XTT reduction assay was used to quantify the effects of chitosan on biofilms produced by *C. albicans*. As shown in Fig. 2, the different phases of *C. albicans* biofilm formation were significantly susceptible to chitosan. At 2 h, the OD values, as determined by an enzyme-labeled instrument, were similar to when the concentration of chitosan was >0.0313%. A statistically significant difference was observed in the OD values between the 0.0313 and 0.0156% chitosan groups (P<0.05). In addition, a marked difference was observed between all the chitosan groups and the positive control (P<0.05). The results did not demonstrate a statistically significant difference between biofilm development at 2 and 8 h, and the optimum concentration of chitosan was almost equal. Compared with the biofilms grown for 2 or 8 h, the OD values for the biofilms grown for 24 or 48 h were significantly higher following exposure to chitosan at a concentration of 0.0313%. The results revealed that biofilms in the mature phase (24-8 h) demonstrated less susceptibility to higher concentrations of chitosan compared with those in the early phase (2–8 h). Thus, biofilm formation exhibits a significant resistance to the antifungal activity of chitosan.

**Figure 3:**
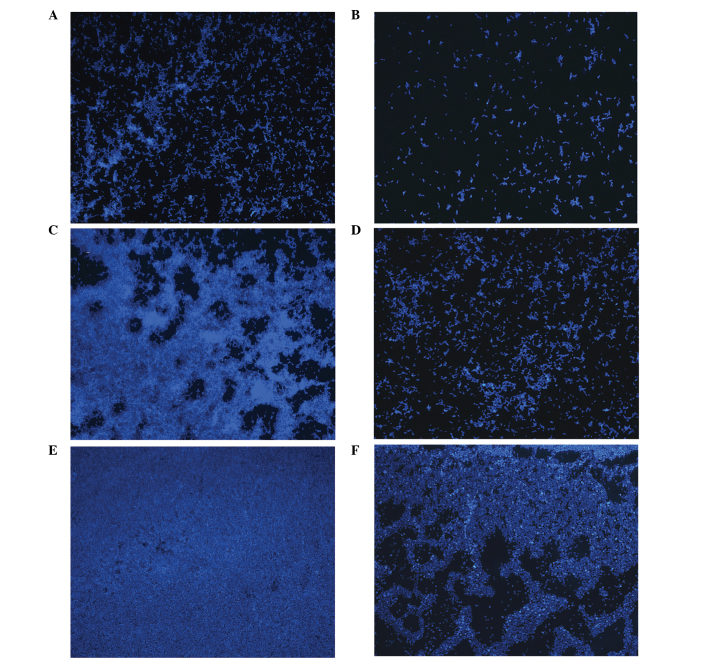
Fluorescence microscopy images showing the three developmental phases of *Candida albicans* biofilms in the (A, C and E) absence or (B, D and F) presence of 0.0625% chitosan (A and B, early phase; C and D, intermediate phase; E and F, maturation phase; magnification, ×10).

A previous study observed that *C. albicans* biofilm formation on polymethylmethacrylate strips progresses in three distinct developmental phases: Early, intermediate and maturation (10). The effect of chitosan on the temporal development of *C. albicans* biofilms on cover slips was investigated with fluorescence microscopy using Calcofluor White M2R, a UV-excitable dye that binds chitin and β-glucan and has long been used to highlight the fungal cell wall (17). Fluorescence microscopy was performed to visualize and compare the three distinct developmental phases between the untreated and chitosan-treated *C. albicans* biofilms (Fig. 3). In the early stage, characteristic microcolonies and short hyphae appeared on the surface of the cover slips of the untreated cells (Fig. 3A). As shown in Fig. 3B, a few yeast cells were dispersed in the 0.0625% chitosan-treated group at the same stage. During the intermediate developmental phase, a dense network of yeast cells surrounded by a large amount of noncellular material was observed in the untreated group, with the metabolically active cells existing in a network (Fig. 3C). The quantity of yeast cells in the chitosan-treated group at the same stage was lower compared with the untreated group, and the cells were unable to congregate and develop into a bioflim (Fig. 3D). In the maturation development phase, the surfaces of the cover slips were coated with a thick layer of yeast cells and noncellular material; however, it was difficult to distinguish between the cell types (Fig. 3E). By contrast, the chitosan-treated group exhibited yeast cells lacking a network structure and a significantly lower metabolic activity compared with the untreated group (Fig. 3F).

Fluorescence microscopy images were used to correlate the XTT reduction assay results with the visual effects of the biofilm formation at the various exposure times. The results indicated that chitosan was not only able to inhibit the metabolic activity of cells in maturing biofilms, but also delay biofilm formation by terminating the fusion process.

### Direct visualization of the effect of chitosan on C. albicans biofilms

**Figure 4:**
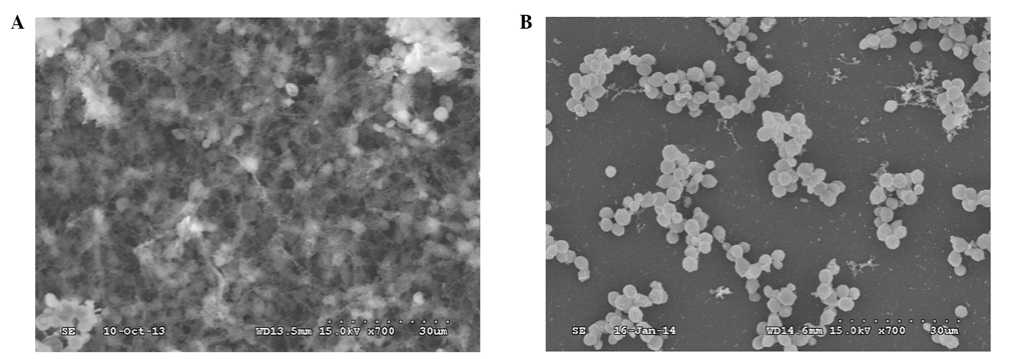
(A) *Candida albicans* biofilms cultured in sabouraud dextrose broth for 24 h showed fungal cells surrounded by large amounts of exopolymeric matrix. (B) By contrast, biofilms co-incubated with 0.0625% chitosan exhibited yeast cells without a capsule, releasing polysaccharides. Scale bar, 30 μm. Maginification, ×700.

SEM examination was used to visualize the structural differences between normal and chitosan-treated *C. albicans* biofilms. Normal *C. albicans* biofilms exhibited a dense network of hyphae surrounded by vast amounts of exopolymeric matrix (Fig. 4A). By comparison, chitosan-treated *C. albicans* expressed a biofilm that lacked a normal network structure and released polysaccharide (Fig. 4B). These results demonstrated that chitosan significantly inhibited *C. albicans* biofilms *in vitro*.

## Discussion

Currently, fungal infections present an increasing threat to the growing number of immunocompromised patients. These contaminations, of which Candidiasis is the most common, represent one of the most prevalent infections in US hospitals (18). A leading causative agent of Candidiasis continues to be *C. albicans* (18). However, numerous problems remain in the current therapies for fungal infections. Antifungal treatment strategies for *C. albicans* are limited to a small armamentarium of compounds, which mainly include azoles (such as fluconazole), polyenes (such as amphotericin B) and echinocandins (19). However, there are clinical drawbacks to these drugs due to their toxicity (20). Therefore, identifying alternative antifungal agents with fewer side effects is required. Furthermore, since the majority of fungal infections are caused by biofilms, this provides an additional reason for the low success rate of treatment. Chitosan offers a flexible, biocompatible platform for designing coatings to protect surfaces from infection and decreasing the metabolic activity and survival rate of *Candida* species biofilms (21). The characteristics of chitosan, including its antiadherent and antifungal properties, mean that it is a strong candidate for treatment against fungal biofilms. In a normal conjunction, the *C. albicans* biofilm proceeds into early, intermediate and maturation phases of development. The extracellular material, as observed by microscopy, is predominantly composed of cell wall-like polysaccharides containing mannose and glucose residues. Biofilms grown on cover slips have a distinct biphasic structure composed of an adherent blastospore layer covered by sparser hyphal elements embedded in a deep layer of extracellular material. The structure that meditates cell interactions with the environment is the cell wall, which may be a viable factor in the adhesion of fungi to solid surfaces.

The present study analyzed the role of chitosan on *C. albicans* using the MIC. The results indicated that chitosan had high fungistatic activity against these planktonic cells. Repeated XTT reduction assay analysis revealed that chitosan at a concentration of 0.0313% had the potential to kill >50% of cells in the early and intermediate phases of biofilm development. However, a higher concentration of chitosan was required to kill cells in mature biofilms. Mature biofilms of *C. albicans* were significantly less susceptible to chitosan than planktonic cells and initial biofilms. The results of the current study indicated that an addition of exogenous chitosan to *C. albicans* biofilms reduced the metabolic activity of cells significantly and apprehended the adhesion of yeast cells to the polystyrene surface. Previous studies have demonstrated that the biofilm phenotype confers resistance to antifungal therapy, which is consistent with the results of the present study (22,23). Physical stress of the biofilm structure due to permeabilization of the cellular membrane, which permits higher levels of penetration by chitosan and a more effective delivery of its antifungal activity, may be the cause of this phenomenon (24,25).

Fluorescence and SEM techniques allowed the morphology and structure of biofilms to be analyzed in the present study. Fluorescence microscopy visualized the gross biofilm morphology and the appearance of the extracellular matrix during biofilm formation. SEM visualized the structure of the biofilms surface without distortion of the native biofilm structure. Progressing in three distinct developmental phases, *C. albicans* biofilm formation is divided into early, intermediate and maturation stages. The results of the present study revealed that in the overall biofilm formation period, the chitosan-treated biofilms exhibited a slower growth compared with those that were untreated. Although chitosan treatment did not completely destroy the yeast cells, the staining observed with fluorescence and SEM demonstrated that chitosan-treated biofilms were significantly inhibited in the formation of a complex network.

In conclusion, the results of the present study demonstrated that chitosan may be developed as an antimicrobial agent against the treatment of clinically-associated fungal biofilm diseases. Although an inhibitory effect of chitosan on biofilm production by *C. albicans* was reported in the laboratory and in clinical isolates *in vitro*, further studies using animal models of pulmonary *C. albicans* infection are required.

## References

[1] Hallahan AR, Shaw PJ, Rowell G, O’Connell A, Schell D, Gillis J (2000). Improved outcomes of children with malignancy admitted to a pediatric intensive care unit. Crit Care Med.

[2] Booy R, Habibi P, Nadel S (2001). Reduction in case fatality rate from meningococcal disease associated with improved healthcare delivery. Arch Dis Child.

[3] Tenner PA, Dibrell H, Taylor RP (2003). Improved survival with hospitalists in a pediatric intensive care unit. Crit Care Med.

[4] Chen Yan-jun WF (2013). Study on pathogens causing lower respiratory tract infections and their drug resistance. Zhong Guo Bing Yuan Sheng Wu Xue Za Zhi.

[5] Adair CG, Gorman SP, Feron BM (1999). Implications of endotracheal tube biofilm for ventilator-associated pneumonia. Intensive Care Med.

[6] Crump JA, Collignon PJ (2000). Intravascular catheter-associated infections. Eur J Clin Microbiol Infect Dis.

[7] Maki DG, Tambyah PA (2001). Engineering out the risk for infection with urinary catheters. Emerg Infect Dis.

[8] Douglas LJ (2003). *Candida* biofilms and their role in infection. Trends Microbiol.

[9] Blankenship JR, Mitchell AP (2006). How to build a biofilm: a fungal perspective. Curr Opin Microbiol.

[10] Chandra J, Mukherjee PK, Ghannoum MA (2008). *In vitro* growth and analysis of *Candida* biofilms. Nat Protoc.

[11] Carlson RP, Taffs R, Davison WM, Stewart PS (2008). Anti-biofilm properties of chitosan-coated surfaces. J Biomater Sci Polym Ed.

[12] Alonso MJ, Sánchez A (2003). The potential of chitosan in ocular drug delivery. J Pharm Pharmacol.

[13] Kulikov SN, Tiurin IuA, Fassakhov RS, Varlamov VP (2009). Antibacterial and antimycotic activity of chitosan: mechanisms of action and role of the structure. Zh Mikrobiol Epidemiol Immunobiol.

[14] Pasquantonio G, Greco C, Prenna M (2008). Antibacterial activity and anti-biofilm effect of chitosan against strains of *Streptococcus mutans* isolated in dental plaque. Int J Immunopathol Pharmacol.

[15] Meshulam T, Levitz SM, Christin L, Diamond RD (1995). A simplified new assay for assessment of fungal cell damage with the tetrazolium dye, (2,3)-bis-(2-methoxy-4-nitro-5-sul- phenyl)-(2H)-tetrazolium-5-carboxanilide (XTT). J Infect Dis.

[16] Tsang PW, Bandara HM, Fong WP (2012). Purpurin suppresses *Candida albicans* biofilm formation and hyphal development. PLoS One.

[17] Albani JR, Plancke YD (1998). Interaction between calcofluor white and carbohydrates of alpha 1-acid glycoprotein. Carbohydr Res.

[18] Wisplinghoff H, Ebbers J, Geurtz L, Stefanik D, Major Y, Edmond MB, Wenzel RP, Seifert H (2014). Nosocomial bloodstream infections due to *Candida* spp. in the USA: species distribution, clinical features and antifungal susceptibilities. Int J Antimicrob Agents.

[19] Georgopapadakou NH, Walsh TJ (1994). Human mycoses: drugs and targets for emerging pathogens. Science.

[20] Ramage G, VandeWalle K, Bachmann SP, Wickes BL, López-Ribot JL (2002). In vitro pharmacodynamic properties of three antifungal agents against preformed *Candida albicans* biofilms determined by time-kill studies. Antimicrob Agents Chemother.

[21] Martinez LR, Mihu MR, Tar M (2010). Demonstration of antibiofilm and antifungal efficacy of chitosan against candidal biofilms, using an in vivo central venous catheter model. J Infect Dis.

[22] Hawser SP, Douglas LJ (1995). Resistance of *Candida albicans* biofilms to antifungal agents in vitro. Antimicrob Agents Chemother.

[23] Mukherjee PK, Chandra J, Kuhn DM, Ghannoum MA (2003). Mechanism of fluconazole resistance in *Candida albicans* biofilms: phase-specific role of efflux pumps and membrane sterols. Infect Immun.

[24] Rabea EI, Badawy ME, Stevens CV, Smagghe G, Steurbaut W (2003). Chitosan as antimicrobial agent: applications and mode of action. Biomacromolecules.

[25] Sudarshan NR, Hoover DG, Knorr D (1992). Antibacterial action of chitosan. Food Biotechnol.

